# Probing in situ capacities of prestressed stayed columns: towards a novel structural health monitoring technique

**DOI:** 10.1098/rsta.2022.0033

**Published:** 2023-04-03

**Authors:** Jiajia Shen, Luke Lapira, M. Ahmer Wadee, Leroy Gardner, Alberto Pirrera, Rainer M. J. Groh

**Affiliations:** ^1^ Department of Aerospace Engineering, Bristol Composites Institute, University of Bristol, Bristol BS8 1TR, UK; ^2^ Department of Civil and Environmental Engineering, Imperial College London, South Kensington Campus, London SW7 2AZ, UK

**Keywords:** structural stability, buckling, virtual testing, on-site assessment, mode interaction, machine learning

## Abstract

Prestressed stayed columns (PSCs) are structural systems whose compressive load-carrying capacity is enhanced through pre-tensioned cable stays. Much research has demonstrated that PSCs buckle subcritically when their prestressing levels maximize the critical buckling load of the theoretically perfect arrangement. Erosion of the pre-tensioned cables’ effectiveness (e.g. through creep or corrosion) can thus lead to sudden collapse. The present goal is to develop a structural health monitoring (SHM) technique for in-service PSCs that returns the current *structural utilization factor* based on selected probing measurements. Hence, PSCs with different cable erosion and varying compression levels are probed in the pre-buckling range within the numerical setting through a nonlinear finite element (FE) model. In contrast to the previous work, it is found presently that the *initial lateral stiffness* from probing a PSC provides a suitable health index for in-service structures. A machine learning-based surrogate is trained on simulated data of the loading factor, cable erosion and probing indices; it is then used as a predictive tool to return the current utilization factor for PSCs alongside the level of cable erosion given probing measurements, showing excellent accuracy and thus provides confidence that an SHM technique based on probing is indeed feasible.

This article is part of the theme issue ‘Probing and dynamics of shock sensitive shells’.

## 1. Introduction

**Figure 1.**
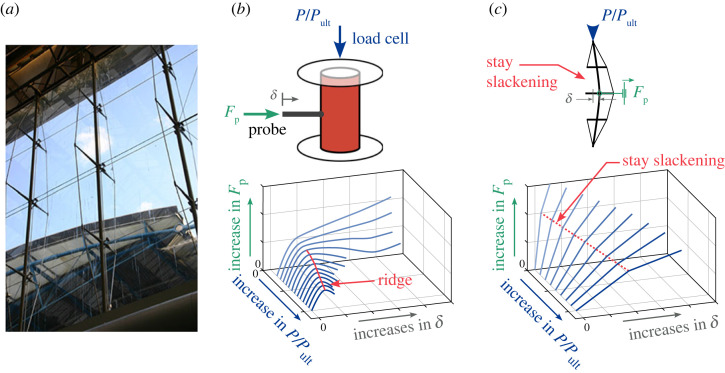
(*a*) Triple-bay PSC used as roof supports in the former Eurostar terminal in Waterloo Station, London. Probing landscape showing equilibrium paths of probe force $F_{p}$ versus probing displacement δ for (*b*) cylinders, and (*c*) PSCs, with varying utilization ratio $P/P_{\text{ult}}$, where $P_{\text{ult}}$ is the current ultimate load-carrying capacity of the structure. For cylinder probing, the maximum points of the probe force–displacement curves are the key indices for non-destructive testing [1]. For probing of PSCs, the first kink in the probe force–displacement curves and the slope of the probing curve before the first kink are the probing indices to estimate the ultimate load. Photograph (*a*) courtesy of Dr Daisuke Saito, diagram (*b*) is adapted from ref. [2]. (Online version in colour.)

Prestressed stayed columns (PSCs), as shown in figure 1, comprise a slender main column and a system of cross arms reinforced by tensioned cables. PSCs are well known for their aesthetic appearance, high efficiency and speed/ease of construction [3–5]. In recent decades, they have been used in many engineering projects, some of which have entered their second or third decades of service [6]. Owing to their slender structural form, their governing failure mode is generally through buckling instabilities [7,8]. Previous works [9–14] have demonstrated that the perceived optimum design, where the critical buckling load is maximized, may lead to dangerously unstable interactive post-buckling behaviour and a high degree of imperfection sensitivity [15]. Commercial FE packages with robust nonlinear solvers can predict the performance of the PSCs accurately in these scenarios [5,7]. However, as far as the authors are aware, little research work has been conducted to assess their performance in-service, i.e. to assess the in situ load-carrying capacity when affected by temporal effects such as structural deterioration from corrosion or loss in prestress from cable creep/relaxation. Considering the potentially severe consequences of a structural failure on public safety and the extent of the intended design lifespan of PSCs, it is, therefore, prudent to develop a robust, efficient and economic structural health monitoring (SHM) technique to assess their performance. The aim of the current work is to develop such a technique based on a new strategy for probing the stability landscape of PSCs.

Most existing non-destructive SHM techniques in civil engineering are based on the measurement of the dynamic characteristics of structures, using ambient or forced vibration methods [16–20]. However, for slender structures governed by buckling instabilities, a testing approach based on vibrations can introduce errors arising from noise, damping and inertia, and therefore fail to provide the required information about the remaining service life of the structure [21]. By contrast, a straightforward approach based on the expected bifurcation landscape of a tested structure, and deviations thereof, may be more suitable for assessing the current in-service utilization factor defined as the ratio of the applied load to the ultimate load.

### (a) Development of the probing methodology

Thompson *et al*. [22,23] proposed a non-destructive technique to estimate the buckling resistance of shells—structures that are notoriously sensitive to initial imperfections—by probing them laterally. The probing force stimulates a single dimple in the shell wall—a deformation mode that has been identified to play a central role in the onset of buckling [24,25]. This technique has been implemented experimentally to assess the load-carrying capacity of axially compressed cylindrical shells [1,2,26]. Specifically, relationships of the probing force $F_{p}$ versus the corresponding displacement δ at different normalized axial compression levels $P/P_{\text{ult}}$ were measured, as shown in figure 1*b* (adapted from [2]). The locus of points connecting the maxima in the probe force–displacement curves—which intuitively describes the stiffness of shells in resisting lateral perturbations—is a fold line; in this case, also called a ‘ridge’ [2,27] owing to the shape of the surface in figure 1*b*. By determining points on this ridge for different levels of axial compression and extrapolating the ridge to the point where the probing reaction force reaches zero, the buckling load of the cylinder being tested can, in principle, be determined [1,2,28]. In this manner, the actual buckling load of an imperfect cylinder can be extrapolated without inducing the unstable buckling event. The accuracy and reliability of ridge tracking in assessing the load-carrying capacity largely depends on the probing location and the imperfection distribution of the tested shells [26]. In practice, a surface scanning device is required to obtain the imperfection profile of the shell and determine the best probing location (or locations), where the sharpest imperfection resides [29,30]. As a result, inadvertent buckling and collapse of the cylinder during a test is possible if the cylinder is probed at the wrong location [1]. Moreover, this probing method, as currently devised, requires variations of the compression level in the tested structure, which would be practically very difficult to achieve in PSCs in-service. Finally, this probing method needs a relatively large probing displacement to reach the ridge, requiring potentially very stiff and bulky apparatus where probe forces themselves could potentially compromise the integrity of the in situ structure—a totally unacceptable scenario.

An important prerequisite of the probing method, which has been generally overlooked by preceding researchers, is the concept of *shape control* over critical modes of instability [31]. Consider a generic structure with a tangential stiffness matrix $K_{T}$ (derived computationally or probed experimentally). At the first instability, this matrix becomes singular with at least one zero eigenvalue and corresponding critical eigenvectors that describe the buckling modes. Hence, if a structure is probed at the point of buckling in a manner that imposes the critical buckling mode, then the resistance measured by the probing actuators will, by definition, be zero. For loads less than the buckling load, the measured resistance will be positive, indicated by a positive slope of the probing force versus probing displacement path or a positive value of the ridge force, and will thus sample a specific stiffness component of the structure in the direction of the applied probing mode. If the induced probing mode does not reflect the buckling mode—i.e. the eigenvector of $K_{T}$ with the smallest associated principal direction of stiffness—then the measured stiffness increases. In essence, the imposed probing mode is a modal combination of eigenvectors rather than purely aligned with the critical eigenvector of smallest principal stiffness. As a result, extrapolating measurements of the ridge force—as is currently being proposed in ridge tracking—will always over-predict the true buckling load unless the appropriate *shape control* is exerted over the structure by the probes.

On the basis of the concept of *shape control* [31], Shen *et al.* [30,32] proposed a methodology to generalize the design of probing strategies to investigate the bifurcation manifolds of generic nonlinear structures. This experimental methodology uses multiple probes, which enables the formulation of the experimental tangential stiffness matrix of the tested structures and the investigation of tortuous bifurcation manifolds of structures with complex post-buckling characteristics. The effectiveness of the technique has been demonstrated numerically with several benchmarks, including a three-bay PSC, where three independent probes, i.e. at quarter-height, mid-height and three-quarter-height, were used to trigger and subsequently control the symmetric and antisymmetric post-buckling modes [33]. However, even though this advanced probing technique has been demonstrated to be successful, robust and able to deliver additional information about the nonlinear structural response, the required procedures may, in general, be too complex or even inappropriate for structures that are in-service. Therefore, modifications to both probing techniques outlined earlier are required for in situ SHM applications.

### (b) Probing as a generic technique for structural health monitoring

In most cases, structural engineers are primarily concerned with the structural utilization factor (the currently applied load divided by the ultimate load-carrying capacity), and also to some extent the overall structural stiffness. Accordingly, and focusing on PSCs, the aim of this work is to develop a simple and reliable non-destructive monitoring technique to establish a relationship between probing measurements and the utilization factor, as well as levels of temporal deterioration. In addition, the goal is to overcome constraints imposed by shape control in successfully applying ridge tracking, thereby minimizing the number of probes required.

Key to the currently proposed approach is the insight that probing allows a tangential stiffness component to be measured that is associated with a certain deformation mode. So long as one or multiple probing measurements are related to the state variables of interest (utilization factor, cable degradation), these tangential stiffness components can be used for health monitoring, as has, for example, been successful in vibration-based SHM [21]. Currently, probing is used at a single location, or sequential probing at multiple locations, to determine individual tangential stiffness components of a three-bay PSC. The measured stiffness components are defined as probing indices and, crucially, need not reflect the tangential stiffness in the direction of the critical buckling mode. As such, tracking of a ridge force to a zero value is not required. Instead, statistical relations between the probing indices and certain state variables are determined by regression using artificial neural nets in a machine learning (ML) surrogate. Once the surrogate has been trained with sufficient data, such as from numerically simulating the effects on a particular PSC from different axial loads, the condition of a PSC in-service, i.e. the utilization factor and the severity of defects, can be identified using discrete probing measurements. Compared with the ridge tracking and experimental continuation approaches, the proposed method greatly simplifies the probing system and procedure. Specifically, instead of multiple probes working simultaneously to achieve shape control, only one probe or a probe pair is used for probing discrete locations sequentially. Moreover, compared to the case of probing for ridge tracking, only the initial probing stiffness is determined, which requires small displacements and, therefore, probing apparatus can be smaller in size and less intrusive perturbations would be required for monitoring purposes.

The remainder of this article is structured as follows. Section 2 presents the problem, the development of the FE model and the probing technique, while in §3, the response of PSCs under probing is presented, and the results are discussed. The implementation of the ML framework, which focuses on predicting the in situ utilization factor of PSCs and the deterioration of the cables, is developed in §4 with results discussed in §5. Finally, conclusions are given in §6.

## 2. Model development and analysis procedure

**Figure 2.**
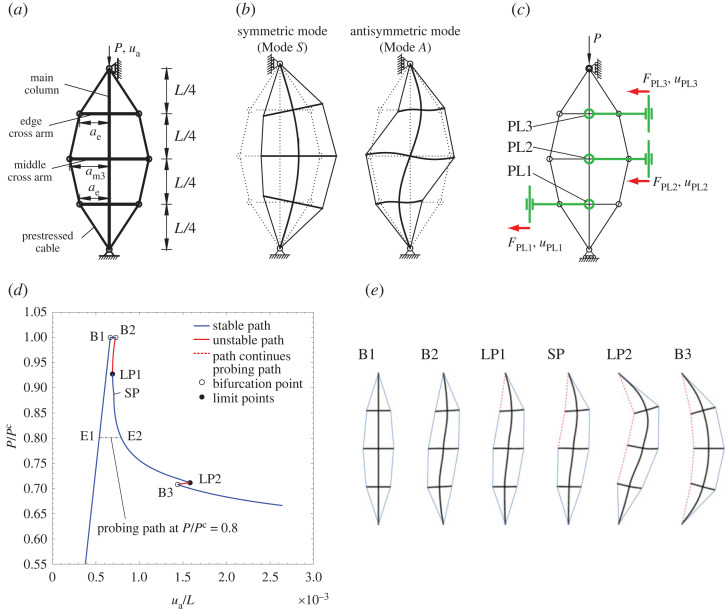
(*a*) Triple-bay PSCs with geometric definitions. (*b*) Symmetric (Mode S) and antisymmetric buckling (Mode A) eigenmodes. (*c*) Probe layout for the triple-bay PSC for the SHM technique. Note that the probes allow rotations and vertical displacements to prevent reaction moments and vertical reaction forces that would perturb the equilibrium path. Note that the probes only represent the location where the PSC is probed. In the present scheme, only one probe or a probe pair is needed for probing these locations sequentially. (*d*) Equilibrium path of the perfect PSC with properties presented in table 1, where $P^{C}$ is the critical buckling load of the PSC and with the labelled stability based on displacement-controlled loading. The full equilibrium manifold diagram can be found in [33]. (*e*) Deformation profiles at selected equilibria labelled in (*d*). (Online version in colour.)

In the current work, a triple-bay PSC under axial compression is considered, as shown in figure 2*a*. The main column member is of length L and is assumed to have a circular hollow cross section with external diameter $\phi_{\text{co}}$ and internal diameter $\phi_{\text{ci}}$. The cross arms are uniformly spaced along the column length with the outer ones being of length $a_{e}$, with $a_{e}<a_{m3}$, where $a_{m3}$ is the length of the middle cross arm. The material and geometric properties of the PSC are based on those in a previous study [33] and are presented in table 1. As shown in the displacement-controlled equilibrium manifold of figure 2*d*, in this configuration, the critical buckling mode of the PSC is antisymmetric, and the initial post-buckling behaviour is weakly stable. A secondary bifurcation point on the post-buckling path, with the newly triggered eigenmode being symmetric, leads to an unstable snap-back response and a sharp drop in the load-carrying capacity [13].

**Table 1.** Material and geometric properties of the prestressed stayed columns.

Young’s modulus of the main column and cross arms	$E_{c}=E_{a}=210\;\mathrm{kN}\;{\mathrm{mm}}^{-2}$
Young’s modulus of the stays	$E_{s}=202\;\mathrm{kN}\;{\mathrm{mm}}^{-2}$
Yield stress of the main column	$f_{y}=355\;\mathrm{MPa}$
Outside diameter of the main column and cross arms	$\phi_{\mathrm{co}}=\phi_{\mathrm{ao}}=38.1\;\mathrm{mm}$
Inside diameter of the main column and cross arms	$\phi_{\mathrm{ci}}=\phi_{\mathrm{ai}}=26.3\;\mathrm{mm}$
Diameter of the stays for triple-bay column	$\phi_{s}=2.5\;\mathrm{mm}$
Length of the main column for triple-bay column	L=5080 mm
Length of the edge cross arm for triple-bay column	$a_{e}=406.4\;\mathrm{mm}$
Length of the middle cross arm for triple-bay column	$a_{m3}=508\;\mathrm{mm}$
Prestress in the cables	$\sigma_{s}=400\;N\;{\mathrm{mm}}^{-2}$

Owing to this subcritical post-buckling behaviour, the PSC can be probed from any point along the fundamental pre-buckling path to assess the energy barrier between fundamental and post-buckling equilibria at the same axial compressive load P. However, the success of the probing technique is strongly dependent on the control that the applied probing forces exert over the overall shape of the structure [30]. Considering the nonlinear interaction between symmetric and antisymmetric modes in the post-buckling range, as shown in figure 2*e*, it was demonstrated in [33] that three independent probes are required to control the coupled deformation mode on the unstable post-buckling path. As a result, three independent probes are ideally needed to determine the ultimate load accurately by ridge tracking in the probing stability landscape. However, owing to the practical complexity involved in synchronizing three probes on-site, a single probe, or at the most, a pair of synchronized probes, is considered hereinafter.

Furthermore, estimating the ultimate compressive load of PSCs using the ridge-tracking probing approach as previously proposed in the literature [2,27] would require variation of the axially compressive load. However, modifying the axial loading of columns in-service is not practical. Therefore, an alternative approach is proposed that does not require the variation of the axial compression level, which, nonetheless, still aims to develop a relationship between key characteristics (defined as *probing indices*) of the probing force–displacement equilibrium curve and the structural utilization factor of PSCs. In addition, the probing indices are correlated with the severity of cable stiffness erosion from, say, corrosion, such that the structural health condition of PSCs can be assessed by probing the main column laterally at selected locations.

### (a) Finite element modelling

The commercial FE software Abaqus [34] is used to model the PSCs. The column and cross arms are modelled with Timoshenko beam elements with linear interpolation functions (‘B21’ in the Abaqus element library). The stays are modelled with two-node truss elements (‘T2D2’ in the Abaqus element library), with the ‘no compression’ option enabled to ensure that the elements can only resist tension. The prestress in the stays is modelled as an *initial condition*. The main column is simply supported; the cross arms are rigidly connected to the main column; and the stays are pinned to the ends of the main column and the cross arms. The static Riks solver [35] was implemented within Abaqus Standard; following a mesh sensitivity study, an arrangement comprising 40 beam elements per column segment divided by the cross arms, 20 beam elements per cross-arm segment and a single truss element per stay has been demonstrated to be sufficiently accurate when verified by comparison with previous analytical [13] and numerical studies [36,37], whose FE models were in turn calibrated against experiments [7].

### (b) Imperfections and in-service performance deterioration

Typically, PSCs in-service have a variety of different imperfections, such as geometric imperfections arising from material production, fabrication and handling during erection; non-uniformity of tension in the stays or uneven settlement at the foundation. Herein, only geometric imperfections are considered such that the feasibility of the presently developed approach is studied without needlessly complicating the discussion, while more intricate details are left for future work. The imperfection profiles are assumed to be affine to the symmetric (Mode S) and antisymmetric (Mode A) linear buckling modes, shown in figure 2*b*, with the size of the imperfections assumed to be of the order of those typically observed in manufactured specimens [15], i.e. L/1000 with L being the column length. Imperfections are introduced in the FE models as geometric perturbations using the *Imperfection keyword.

The deterioration of the cables with time (e.g. through creep or corrosion) is represented by a reduction in the initial prestress $T_{\text{in}}$ within stays as well as by the loss of stay stiffness $E_{s}A_{s}$, which is implemented through a reduction in stay cross-sectional area $A_{s}$. Instead of modelling the deteriorating effects across time, a series of FE models are developed with the reduced initial prestress and stay stiffness applied directly, i.e. through a parametric approach. It is found that the loss in the effective area of the cables is the most crucial parameter among the two presently considered in terms of loss in load-carrying capacity when compared with the original configuration. Thus, the remainder of this study focuses on probing of PSCs with reduced stay cable areas, while details of the numerical study of the deterioration of prestress can be found in the electronic supplementary material. As indicated earlier, a more realistic analysis on probing PSCs in-service, considering non-uniform deterioration in combination with other deterioration factors, is left for future work.

**Figure 3.**
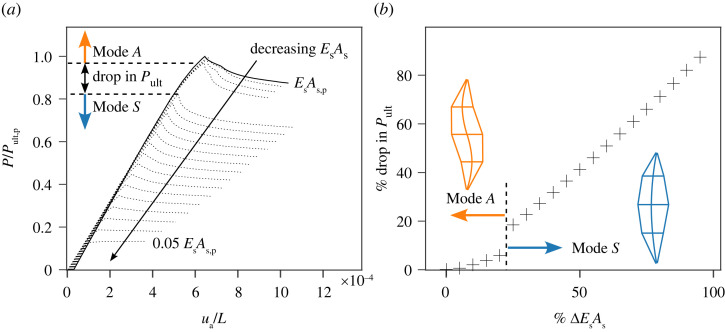
Response of PSCs with different cross-sectional areas of stays $A_{s}$ representing a loss of stay stiffness from corrosion; $A_{s,p}$ corresponds to the case without any erosion in properties, i.e. $\phi_{s}=2.5\;\mathrm{mm}$. (*a*) Equilibrium diagram of axial force against vertical displacement of the top-node normalized by PSC length $u_{a}/L$ for the different configurations. (*b*) Sensitivity of the ultimate load-carrying capacity drop to the stay stiffness drop with the drop in peak ultimate capacity normalized with $P_{\text{ult},p}$, the ultimate load for a PSC with uncorroded cables. Note that the geometry and material parameters of PSCs are the same except for the stay diameters. (Online version in colour.)

The results from the parametric study on reducing the stay cross-section area (from the full area without erosion $A_{s,p}$ to $0.05A_{s,p}$) in all cables are shown in figure 3. Each curve in figure 3*a* represents a PSC with a reduction in stay stiffness $E_{s}A_{s}$ from the initial case $E_{s}A_{s,p}$. For each PSC geometry considered, a sensitivity study was conducted to determine the combination of the symmetric and antisymmetric buckling modes, based on previous interaction models presented in [10], that lead to the lowest ultimate load, and the corresponding equilibrium paths are plotted in figure 3*a*. The drop in peak load-carrying capacity, $P_{\text{ult}}$, shown as a function of loss of stay stiffness is presented in figure 3*b*. As depicted in figure 3*b*, as the stay area reduces, the critical buckling mode of the PSC changes from antisymmetric (Mode A) to symmetric (Mode S). At the transition, the change in mode coincides with a sharp drop in $P_{\text{ult}}$. This change in the buckling mode is caused by the stays not being able to provide sufficient resistance against the column mid-span from deflecting laterally, a factor that has been discussed extensively in previous works [13,15].

### (c) Probing analysis procedure

**Figure 4.**
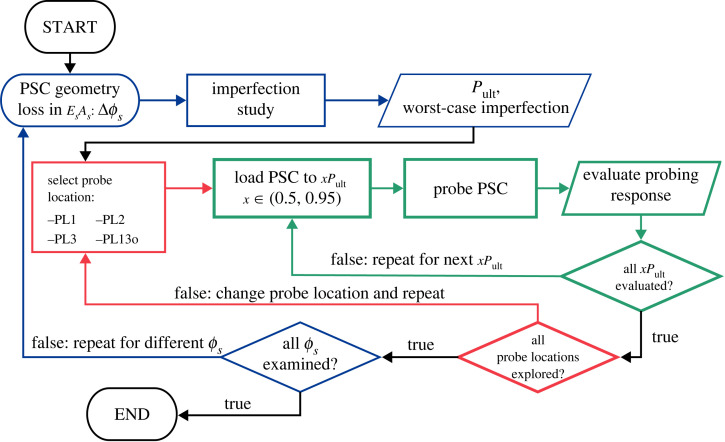
Analysis procedure for determining the probing landscape of PSCs with respect to the loss in cable stiffness $E_{s}A_{s}$ under different axial compression levels. The results of the extensive parametric study is used as the training and verification dataset for the machine learning model. (Online version in colour.)

To establish the relationship between probing indices, drop in cable stiffness and in situ load-carrying capacity, a comprehensive parametric analysis is conducted. The analysis procedure is depicted in figure 4, showing how the probing landscape is explored. Specifically, the ultimate capacity $P_{\text{ult}}$ of a PSC with different $E_{s}A_{s}$ levels (by varying stay diameter $\phi_{s}$) is examined with probes applied at different locations along the main column and under varying levels of $P/P_{\text{ult}}$, where P is the applied axial load on the PSC and $P/P_{\text{ult}}$ is the utilization factor.

To capture the potential mode interaction in the probing path, a combination of symmetric and antisymmetric eigenmodes from the linear buckling analysis are introduced as geometric perturbations in the FE model. A sensitivity analysis on the size and profile of the perturbations is conducted to ensure that the resulting equilibrium paths are physically meaningful, i.e. they represent the minimum energy solution that is most likely to occur in practice and that no more than one negative eigenvalue is detected in the stiffness matrix.

Within the parametric study, nonlinear analyses using the ‘*Static, Riks*’ solver within Abaqus are first conducted to determine the imperfection sensitivity of the PSC configuration under consideration, i.e. its worst-case imperfection mode and the corresponding $P_{\text{ult}}$. Having established $P_{\text{ult}}$, a separate multi-step analysis ensues. The first loading step utilizes the ‘*Static, general*’ solver accounting for nonlinear geometric effects to establish the equilibrium path of the PSC until the service load is achieved. Subsequently, the ‘*Static, Riks*’ solver is employed to trace the nonlinear equilibrium paths of the PSC under different probing forces.

## 3. Response under different probing locations

### (a) ‘Perfect’ PSC

In the current section, the focus is on the behaviour of ‘perfect’ PSCs (with no geometric imperfections and no loss of stiffness in the cables) under probing to reveal their mechanical response, which is quite distinct from the well-studied shell systems. As shown in figure 2*c*, probes providing purely horizontal loading (force or displacement) are applied at the 1/4 (PL1), 1/2 (PL2) and 3/4 (PL3) positions of the column height. Apart from probing the PSC at each of these locations separately, an additional scheme is envisaged where a pair of probes at L/4 and 3L/4 is employed that apply loading in opposite directions (PL13o). The latter scheme is required to probe in the direction of the critical buckling mode of perfect PSCs (antisymmetric mode). Note that the probing scheme is different from the multiple-probe scheme in the authors’ preceding work [33]. Here, only one probe or a probe pair is needed, and each location is probed sequentially.

**Figure 5.**
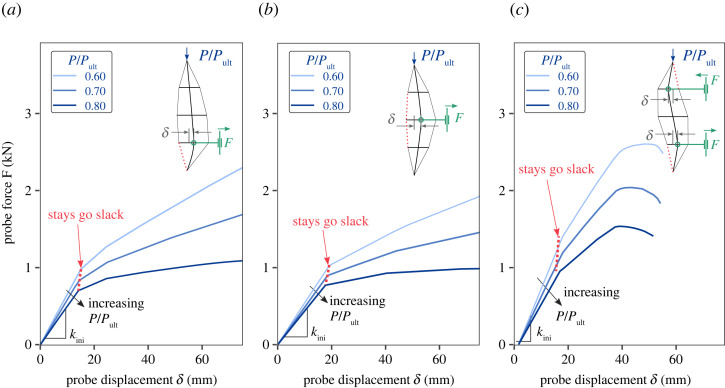
Probing equilibrium paths for perfect PSCs at different axial compression levels $P/P_{\text{ult}}$ (set before the application of the probing force). Note that the axial compression force applied to the PSC remains constant during probing. Probing layouts: (*a*) PL1, (*b*) PL2 and (*c*) PL13o. The first drop in stiffness coincides with the indicated stays going slack (see the dashed cable of PSCs in the insets). The initial stiffness response of the PSC to probing $k_{\text{ini}}$ is also depicted. (Online version in colour.)

Figure 5 presents the probing equilibrium paths under different axial compression levels (namely, 60%, 70% and 80% of the ultimate load of the perfect PSC, $P_{\text{ult},p}$), when the PSC is probed with layouts PL1, PL2 and PL13o, respectively. Generally, the probing equilibrium paths are piecewise linear and divided by kinks that correspond to individual stays slackening on the concave side of the overall deformation shape, as depicted by the dashed lines in the respective insets. The slackening of the cables leads to a drop in the lateral stiffness of the PSC. For low levels of axial compression ($P/P_{\text{ult}}=0.6$), the PSC resists the lateral probing with positive stiffness. For greater levels of compression, the stiffness after the first or second kink may become negative. In the probing approaches previously proposed for shells, the peak of the probing equilibrium path, the ‘ridge’ curve in figure 1*b*, is used as an index to reflect the energy barrier between the pre- and post-buckling equilibrium states. The equilibrium landscape is explored with a single probe while varying the external compressive loads. As discussed previously, altering in-service loads is not practically feasible in the real world. Moreover, since the post-buckling response of PSCs can be interactive particularly when the critical mode becomes antisymmetric [13–15], and at least three independent probes are required to impose and exert sufficient control authority on their deformation [33]; therefore, the established single-probe approach [1,2,28] cannot be adopted directly. In addition, reaching the ridge can require a relatively large probing displacement or forcing magnitude if the lateral stiffness of the structure is large, which, in turn, calls for appropriately sized, and hence expensive, probing systems.

**Figure 6.**
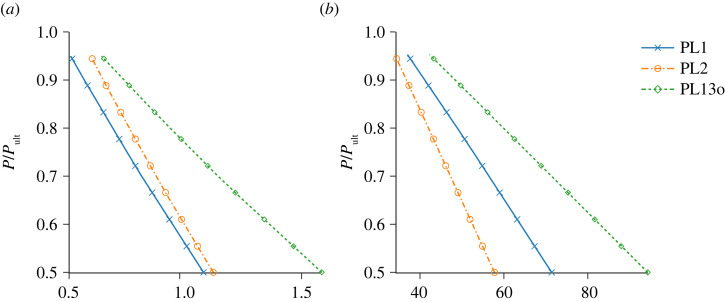
Relationship between the different axial compression levels $P/P_{\text{ult}}$ versus (*a*) probe force (in kN) when the stay goes slack and (*b*) the initial stiffness (in N/mm) both for the ‘perfect’ PSC with $\phi_{s}=2.5\;\mathrm{mm}$. (Online version in colour.)

Figure 6 depicts the relationship between the two proposed probing indices and the utilization factor. Figure 6*a* presents the probe force at the first kink (where the first cable becomes slack), while figure 6*b* presents the initial (lateral) stiffness of the probing equilibrium path with both graphs being plotted versus the utilization factor $P/P_{\text{ult}}$ for three different probing schemes. Note that an upper limit of $P/P_{\text{ult}}=0.95$ is set, which is considerably beyond the expectations for in-service structures that are designed using limit state design methods. Generally, the two probing indices, the force at the first kink and initial lateral stiffness, decrease monotonically with an increase in $P/P_{\text{ult}}$.

In the previous work on probing of axially compressed cylindrical shells, the maximum stationary point in the probing force–displacement curve was used as a probing index [2]. The locus of these stationary points for increasing axial compression is the aforementioned ridge and is expected to intersect the zero probe force axis at the buckling point. By definition, the shell provides zero stiffness to lateral probing at the buckling point because a localized force excites the single-dimple buckling mode, which has no associated stiffness. For the PSC considered presently, only the probe layout PL13o is expected to show this characteristic behaviour as it deforms the PSC in a mode sufficiently aligned with the asymmetric eigenmode. Indeed, the authors’ previous work has shown that the initial stiffness for PL13o shown in figure 6*b* reaches zero for $P/P_{\text{ult}}=1$ [33]. This is not immediately evident from figure 6*b* as the quasi-linear trend becomes highly nonlinear in the proximity of the critical point [27].

For a set compression level, the PSC exhibits distinct behaviours for different probing locations, reflecting the lateral stiffness variation at the particular locations. Specifically, the probing force required to reach the first kink is largest for PL13o and smallest for PL1. As for the initial slope, PL13o corresponds to the greatest resistance to probing, while PL2 corresponds to the least resistance. Note that the difference between each case is more pronounced for the initial stiffness plot compared with the force at the first kink. Therefore, it is possible to estimate $P/P_{\text{ult}}$ by probing at different locations and measuring the two probing indices: (1) force at the first kink and (2) the initial lateral stiffness to probing. Compared with the traditional probing approach, where the probing location remains unchanged, and the compressive load level applied on the structure is varied to obtain a sufficient number of data points for regression, the approach proposed herein is adapted to be more convenient for in-service SHM purposes. In light of the authors’ experimental experience on probing nonlinear structures [32], measuring the slope of the probing path is less sensitive to noise since it can be approximated from multiple data points and least-squares regression. By contrast, measuring the force at the kink is more sensitive to noise as it relies on a single data point. Therefore, the initial probing stiffness before the first kink is chosen to be the key probing index for the remainder of the present study.

### (b) Imperfect PSCs with cable area erosion

Section 2(b) shows that the erosion of the cable area leads to a significant drop in the load-carrying capacity alongside a qualitative change in the failure mode of the PSC. In the current section, the probing response of PSCs with different erosion levels in the cables and geometric imperfections as described in §2 are studied. For simplicity, it is assumed that erosion is the same in all cables. Since there is a significant load drop when the erosion in $E_{s}A_{s}$ reaches 22.5% (see figure 3*b*), the present focus is on $E_{s}A_{s}$ erosion that is less than 22.5%. A more detailed analysis considering different erosion rates for each cable is left for future work.

**Figure 7.**
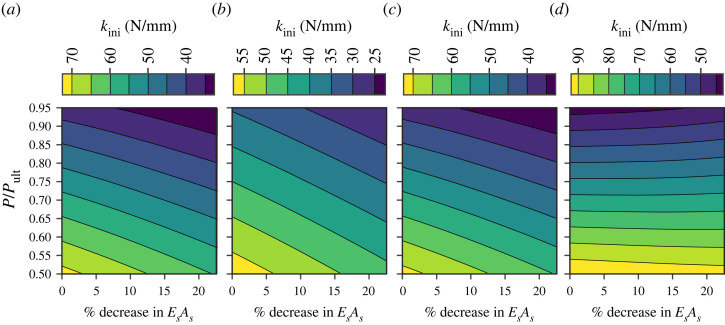
Contour plots of the initial probing stiffness $k_{\text{ini}}$ of imperfect PSCs with varying $E_{s}A_{s}$ and $P/P_{\text{ult}}$, with probing layouts: (*a*) PL1, (*b*) PL2, (*c*) PL3 and (*d*) PL13o. (Online version in colour.)

Generally, as $E_{s}A_{s}$ drops, the initial probing stiffness decreases, as shown in figure 7. This confirms that the initial probing stiffness is an appropriate probing index to assess the in situ capacities of the PSCs. For perfect PSCs, the utilization factor $P/P_{\text{ult}}$ can be estimated by probing in a single location, since the probing index corresponds to a unique $P/P_{\text{ult}}$ value. For imperfect PSCs with a uniform erosion in $E_{s}A_{s}$, two probing measurements are required to infer the utilization factor $P/P_{\text{ult}}$ and the erosion in the cables (%$E_{s}A_{s}$). In the following section, a machine learning (ML) framework is developed to establish a link between measured initial probing stiffness at two locations and the current utilization factor $P/P_{\text{ult}}$ and the percentage erosion of $E_{s}A_{s}$ in the cables. While the ML algorithm is first trained by conducting probing simulations at different $P/P_{\text{ult}}$ levels, the application of the ML model for structures in-service effectively inverts the process. By measuring the initial probing stiffness at two locations, both the utilization factor and the erosion of the cables can be inferred.

## 4. Machine learning framework

Note that the initial probing stiffness before the first kink corresponds to a specific component of the experimental tangential stiffness defined by the authors’ preceding work on experimental continuation [32]. Under the experimental continuation framework, an experimental tangential stiffness matrix with reasonable fidelity (i.e. having a sufficient number of probes with independent shape control authority) can describe the bifurcation properties of the tested structure well. System identification through a computational or experimentally measured tangential stiffness matrix of structures has been one of the fundamental methods for SHM techniques [21]. However, the discrete experimental tangential stiffness matrix from experimental continuation does not provide intrinsic information about the current utilization factor or possible damage within the structure. Hence, even with an experimental tangential stiffness matrix of sufficient fidelity, a system identification algorithm is required to establish the relationship between key structural performance variables, i.e. the utilization factor and the location and severity of the structural defects, and certain stiffness components. Presently, the plan is to establish a relationship between discrete tangential stiffness components (probing indices) and state variables, such as the utilization factor and the location and severity of structural defects, by exploiting a ML algorithm.

It was identified in §3 that the probing index of the lateral probing stiffness before the first kink uniquely correlates with the utilization factor $P/P_{\text{ult}}$ of the perfect PSC. For PSCs with cable erosion, $P/P_{\text{ult}}$ can be uniquely estimated using the probing index measured at two different probing locations. In the idealized scenarios modelled herein (with all cables eroding uniformly and only geometric imperfections existing), a simple curve can be fitted to determine a one-to-one relationship between $P/P_{\text{ult}}$ and the probing index. However, for PSCs in practice, many more variables affect the load-carrying capacity including non-uniform erosion levels between cables and loading imperfections. Indeed, a scenario may be envisaged where different combinations of erosion levels between different cables lead to the same probing index at a single location. As a result, the response surface between the probing index, $P/P_{\text{ult}}$, and cable erosion would become more complex. Although many different means for building this response surface exist, a ‘proof-of-concept’ framework based on ML is presented herein.

From the authors’ perspective, a practical and pragmatic probing scheme should satisfy the following requirements:

(i) being insensitive to environmental noise and imperfections;
(ii) the necessary probing force being as small as feasible while sufficiently large to be measured reliably using practical sensors;
(iii) being simple and straightforward to install.

In consideration of the aforementioned criteria, the methodology that is proposed presently is to probe PSCs at the most practically accessible locations on-site; namely, L/4 and L/2. As shown, only a small probe force or displacement is required at these locations to determine the initial probing stiffness accurately.

An artificial neural network (ANN) is developed for the ML model. The probing indices, i.e. the slope of the probing equilibrium path before the first kink measured at L/4 and L/2, are the inputs submitted to the ANN. After an initial study exploring the hyper-parameter space, the ANN is set up with three hidden layers, the first two comprising 20 neurons each and the third layer comprising 10 neurons. Two outputs are generated from the ANN, with the axial stiffness of the cables $E_{s}A_{s}$ determined after the second layer and the utilization factor $P/P_{\text{ult}}$ established after the third layer.

The ANN is developed using TensorFlow with the high-level API Keras to develop the sequential model described [38]. To train the ML model, a total of 310 data points were generated from the FE simulation results at each probing location, by varying $P/P_{\text{ult}}$ in 10 increments in the range [0.5,0.95], and 31 increments of $E_{s}A_{s}/E_{s}A_{s,p}$ in the range [0.775,1.0]. The results from probing the PSC at two locations were then split into a test set comprising 70% of the results to train the ML model, with the remaining 30% used to validate the training model as an unbiased test set. The ‘70–30’ split is executed with a random seed, using the *train_test_split* function in scikit-learn [39]. The hyper-parameter space of the ANN model was explored in detail and examined using the *Adam* solver in TensorFlow with a learning rate of 0.001, a rectified linear unit activation function and a mean absolute error loss function [38].

**Figure 8.**
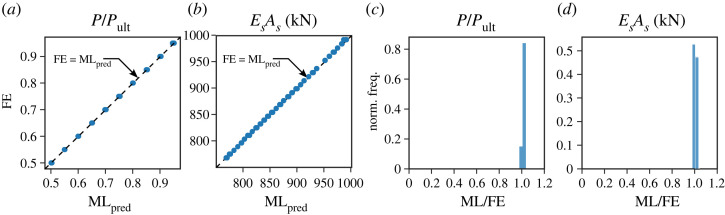
Using the probing layouts PL1 and PL2 to predict $P/P_{\text{ult}}$ (*a*,*c*) and $E_{s}A_{s}$ (*b*,*d*), where 30% of the FE simulation results are compared with their corresponding ML predictions in (*a*,*b*), and the normalized frequency distributions of the ML results normalized by the FE results are shown in (*c*,*d*). (Online version in colour.)

Figure 8*a* presents the utilization factor $P/P_{\text{ult}}$, as determined from the FE simulation results, on the ordinate compared with the prediction from the ML model on the abscissa. Figure 8*c* presents a frequency distribution of the ML results normalized by the FE results, where a value of unity on the abscissa indicates a perfect prediction. The results show excellent correlation, with the distribution concentrated around a value of 1, indicating the accuracy of the approach. The axial stiffness of the stays $E_{s}A_{s}$ is also predicted with great accuracy, as shown in figure 8*b*,*d*, achieved through fine-tuning of the ANN and ML hyper-parameters. Furthermore, the prediction should, ideally, be enforced to always err on the safe side. This can be achieved by adopting physics-informed ANN [40] and will form part of the future work. Such work will also include a sensitivity study on the recommended sample size required to generate a sufficiently accurate ML model for implementation in practical scenarios. Currently, the ML framework is demonstrated to work very well where the reasons for structural deterioration are distinct and well defined. Only extensive further research will be able to demonstrate whether the methodology is reliable when several deteriorating factors interact, but the signs are positive at present. Finally, it is worth noting that the ML model based on other input probing layouts (e.g. PL2 and PL13o), alongside ML models utilizing the probing force at the first kink as input parameters, was also explored, but no performance improvement was observed.

## 5. Discussion

The traditional probing technique was initially developed for axially compressed shells [22], where the ultimate load of the structure is estimated based on probing the structure at various compression loading levels. By contrast, the adapted probing method developed herein tackles the inverse problem; determining the in situ utilization by probing the structure at different locations at the same level of the axial load. The latter is particularly important for inferring the structural utilization of a member in-service, where adjustments to the in situ loading level are simply not practical. In addition, by not probing up to the maximum probing force point (the ridge), the approach of using the initial probing stiffness as a probing index requires much lower levels of probing force, and testing rigs only of a limited size would be necessary. More importantly, the proposed method has a clear physical meaning from the perspective of system identification through the evaluation of the experimentally measured tangential stiffness components of the tested structure. Therefore, the proposed technique is of general purpose and can in principle be used for the non-destructive assessment of any structural form; the fidelity can be improved by increasing the number of probing locations on the tested structure.

Currently, the accuracy of the ML estimation of the utilization factor $P/P_{\text{ult}}$ and cable erosion from measurements of probing indices is entirely based on extensive simulations using a nonlinear FE model to train the ML model. The accuracy of the numerical simulations is therefore crucial for the success of the proposed method and would benefit from the development of a ‘digital twin’ in the future; this would be achieved through an extensive experimental testing programme covering a variety of different erosion levels, such that the ML model is trained with real-world, as well as simulated, data. Moreover, continual SHM techniques, for instance, those used to detect visible defects and imperfections, alongside those using vibration-based techniques to quantify their severity, can be used to enhance the quality of the ‘digital twin’ further. In addition, the present work assumes that the probing system is infinitely stiff, i.e. only the PSC deflects upon probing; this will of course need to be considered in future work using either in situ optical measurements or, perhaps, by accounting for this differential deflection in the ML model.

It should be stressed that the actual loading on in-service structures often varies owing to dynamic effects from the surrounding environment even over short-time scales. A robust noise filtering technique would therefore be needed to eliminate these effects; such techniques have been developed for vibration-based SHM [21] and, in principle, similar techniques could be developed for the quasi-static probing approach.

As noted in figure 4, the generation of each data point requires the creation of a new FE model, which consumes the majority of the current computation time. Moreover, the number of parameters affecting the probing indices would be much larger than currently assumed and the simulated data would need to represent a statistically relevant sample across the distribution of each parameter. Exploring the parameter space using the nonlinear static solver in Abaqus would therefore be time consuming. To generate the simulated data in a more efficient way, a generalized path following solver [41] could be adopted to trace any parameter influencing the structure on a multi-dimensional solution manifold.

From the perspective of the general framework of SHM [42,43], the proposed probing system provides an alternative technique for system identification and decision-making, which hitherto has primarily relied on structural dynamics. The existing mature and widely accepted techniques for data collection, transmission, storage, noise filtering and initial processing can and should be integrated with the proposed method for its practical implementation.

## 6. Conclusion

The response of a three-bay PSC susceptible to complex mode interaction has been explored using the probing concept. The influence of geometric imperfections and material deterioration were considered using a nonlinear FE model developed within the commercial package Abaqus. The characteristic response of the perfect PSC to probing under different compression levels was investigated. Several probing indices reflecting the load-carrying capacity of PSCs were proposed to replace the maximum probe force initially considered for similar techniques applied to shells. An extensive parametric study was conducted to establish how these probing indices evolved with axial compression levels and the erosion in stay stiffness. Based on the numerical results, a SHM method assisted by a ML algorithm based on the probing technique was proposed. It was demonstrated that the trained ML model can provide an accurate prediction of the structural utilization factor and the erosion in the cable stiffness of PSCs based on the probing indices at two probing locations.

Compared with preceding probing techniques (ridge tracking and experimental continuation), the proposed method is based on system identification through certain measured tangential stiffness components of the tested structure. The key innovations and advantages are as follows: (1) ‘shape control’ is no longer a requirement, i.e. the structure does not need to be probed in a deformation mode that is true to the critical buckling mode and which may need the action of multiple coordinated probes, and instead of using multiple probes, a single or multiple sequential probes are used at multiple locations; (2) only a relatively small probing force and displacement are required to obtain the probing indices (essentially providing the initial stiffness of the probing path) that reflect discrete tangential stiffness components; (3) regression through ML is adopted to establish the relationship between probing indices and the utilization factor of in-service PSCs alongside the erosion levels in their pre-tensioned cables.

Future work will develop a theoretical framework considering effects that couple between the probing system and the monitored structure arising from the finite stiffness of the probing system. In addition, the optimum layout of probe locations and the least number of required probes will be investigated further, alongside extending the ML framework to account for further environmental factors and associated uncertainties. Physical testing will also be conducted for validating the theoretical framework alongside the effectiveness of the ML model. The ultimate aim is to develop a health monitoring system that accurately returns the in-service structural utilization factor of PSCs based on quasi-static probing techniques.

## Data Availability

The supporting data are available from this link: https://tinyurl.com/PPSCdata. The data are provided in the electronic supplementary material [44].
